# Non-additive activity modulation during a decision making task involving tactic selection

**DOI:** 10.1007/s11571-021-09702-0

**Published:** 2021-07-30

**Authors:** Wilhelm Braun, Yoshiya Matsuzaka, Hajime Mushiake, Georg Northoff, André Longtin

**Affiliations:** 1 Institut für Genetik, Neural Network Dynamics and Computation, Universität Bonn, Kirschallee 1, 53115 Bonn, Germany; 2 Department of Physics and Centre for Neural Dynamics, University of Ottawa, 150 Louis-Pasteur Pvt, Ottawa, K1N 6N5 Canada; 3 Mind, Brain Imaging and Neuroethics Research Unit, University of Ottawa Institute of Mental Health Research, Royal Ottawa Mental Health Centre, 1145 Carling Avenue, Ottawa, K1Z 7K4 Canada; 4 Division of Neuroscience, Faculty of Medicine, Tohoku Medical and Pharmaceutical University, 1-15-1 Fukumuro, Miyagino ward, Sendai, 983-8536 Japan; 5 Department of Physiology, Graduate School of Medicine, Tohoku University, Aoba Ward, Sendai, 981-8558 Japan

**Keywords:** Decision making, Tactic selection, Data analysis, Prefrontal cortex, Single unit activity, Spiking activity, Non-additivity

## Abstract

Human brain imaging has revealed that stimulus-induced activity does generally not simply add to the pre-stimulus activity, but rather builds in a non-additive way on this activity. Here we investigate this subject at the single neuron level and address the question whether and to what extent a strong form of non-additivity where activity drops post-cue is present in different areas of monkey cortex, including prefrontal and agranular frontal areas, during a perceptual decision making task involving action and tactic selection. Specifically we analyze spike train data recorded in vivo from the posterior dorsomedial prefrontal cortex (pmPFC), the supplementary motor area (SMA) and the presupplementary motor area (pre-SMA). For each neuron, we compute the ratio of the trial-averaged pre-stimulus spike count to the trial-averaged post-stimulus count. We also perform the ratio and averaging procedures in reverse order. We find that the statistics of these quantities behave differently across areas. pmPFC involved in tactic selection shows stronger non-additivity compared to the two other areas which more generically just increase their firing rate pos-stimulus. pmPFC behaved more similarly to pre-SMA, a likely consequence of the reciprocal connections between these areas. The trial-averaged ratio statistic was reproduced by a surrogate inhomogeneous Poisson process in which the measured trial-averaged firing rate for a given neuron is used as its time-dependent rate. Principal component analysis (PCA) of the trial-averaged firing rates of neuronal ensembles further reveals area-specific time courses of response to the stimulus, including latency to peak neural response, for the typical population activity. Our work demonstrates subtle forms of area-specific non-additivity based on the fine variability structure of pre- and post-stimulus spiking activity on the single neuron level. It also reveals significant differences between areas for PCA and surrogate analysis, complementing previous observations of regional differences based solely on post-stimulus responses. Moreover, we observe regional differences in non-additivity which are related to the monkey’s successful tactic selection and decision making.

**Supplementary Information:**

The online version contains supplementary material available at 10.1007/s11571-021-09702-0.

## Introduction

The relationship between spontaneous brain activity and task-evoked activity has generally been assumed to be linear ( Fox et al. 2006, 2007; Sylvester et al. 2009; Fox and Raichle 2007; Arieli et al. 1996; Becker et al. 2011; Azouz and Gray 1999). This means that the activity evoked by task or stimulus is assumed to superpose in a merely additive way on the ongoing spontaneous activity level. However, the generality of such a model of superposition or additive rest-stimulus interaction (Northoff et al. 2010) has recently been put into doubt (He 2013; Huang et al. 2017; Ding and Simon 2014; Ponce-Alvarez et al. 2015; Lynch et al. 2018; Wainio-Theberge et al. 2021). Brain imaging studies on the regional level of neural activity demonstrated non-additive interaction with higher pre-stimulus activity level leading to lower (rather than higher) post-stimulus activity changes, and vice-versa (He 2013; Huang et al. 2017; Ponce-Alvarez et al. 2015; Lynch et al. 2018; Cole et al. 2016; He 2013). The central role of pre-stimulus activity has also been demonstrated on the cellular level in rats (Haslinger et al. 2006; Kisley and Gerstein 1999; Curto et al. 2009) and mice (Llinás et al. 2002; Guo et al. 2015; Pachitariu et al. 2015), and monkeys (van Vugt et al. 2018).

In this study, in contrast, we work exclusively on the single-neuron level. We use data from non-human primates to study how the interaction between pre- and post-stimulus spiking activity (also referred to below as pre- and post-cue activity) relates to sophisticated cognitive behavior, namely the selection of different tactics. We addressed the question whether non-additivity between pre-stimulus and stimulus-induced activity can also be observed on the cellular level in an ensemble of single neurons involved in cognitive dynamics in a higher mammal and how it impacts behavioral performance (Marcos et al. 2013). We performed our analysis at the single neuron level, as opposed to the level of multi-unit activity done by van Vugt et al. (2018) and others. In this way, we do not average out the diversity of the activity of individual neurons.

The stimulus-response relationship in neurons involved in a cognitive task such as working memory (Shafi et al. 2007) can be very rich, displaying a wide array of features across a population of cortical cells. The earlier work by Tsodyks et al. (1997) had in fact already demonstrated a paradoxical effect in neural circuitry where increasing the input to interneurons that inhibit a second interneuron population leads to an increase in activity of this second population. Different cells display different levels of firing rate variability, but also different polarities of firing rate change. Those seminal studies and many others that it spawned point to the importance of characterizing the different classes of cells based on their firing statistics, and in particular the trial-to-trial variations of activity. It has also motivated studies of the kind of dynamics that can explain the variability of transient and steady state activity, e.g. in terms of the structure of recurrent architecture (Bondanelli and Ostojic 2020). Another study (Liang et al. 2018) of medial prefrontal cortex (mPFC) has shown distinct ON and OFF ensembles of neurons that need to be activated during social exploratory behavior, highlighting again the necessary variability of neural activations including the presence of ensembles that respond in an opposing manner.

While additivity comes in basically only one form, where a constant positive fraction of the stimulus-induced activity simply adds to pre-stimulus activity, non-additivity can manifest itself in many forms. For example, a weak form of non-additivity is seen when the increase in activity induced by a same stimulus is a decreasing function of the pre-stimulus activity (He 2013). A high pre-stimulus activity will then increase only marginally in response to the stimulus compared to the case of a low pre-stimulus activity. A stronger form of non-additivity involves e.g. an inverse relationship where the stimulus can actually decrease the activity of the cellular population (He 2013; Huang et al. 2017). Whether non-additivity holds on the single neuron level and, if so, which form it can take and what behavioral correlates it entails remain open issues. To address this question in the context of this strong form of non-additivity, which could in fact be termed subtractivity or suppression, is the goal of our study.

Specifically, in more mathematical terms, we examine the notion of non-additivity on the level of a single trial. Let $N_{\text {pre}}$ and $N_{\text {post}}$ denote integer spike counts in the pre- and post-stimulus intervals, respectively, for one trial in one neuron. One might expect the general relationship $N_{\text {post}}=F(N_{\text {pre}},S)$, i.e. $N_{\text {post}}$ is a perhaps nonlinear function of $N_{\text {pre}}$ and the stimulus *S*. To guide our thinking throughout our paper, we assume for simplicity that $N_{\text {post}}= N_{\text {pre}} + B(N_{\text {pre}})$ where the second term *B* is a positive or negative integer that may depend on the pre-stimulus activity and which accounts for the effect of the stimulus, knowing that more complex relationships may be at work. The additive case corresponds to $B(N_{\text {pre}})=B$ with *B* a positive integer. All other cases can be referred to as “non-additive” to varying degrees. For example, in a supra-additive case *B* increases with $N_{\text {pre}}$, while in the sub-additive case *B* decreases with $N_{\text {pre}}$; both these cases are “non-additive”. A stronger form of non-additivity that concerns us in this paper occurs when $B<0$, meaning that the spike count decreases on a particular trial in a manner that may depend on $N_{\text {pre}}$. The statistics Q and R defined below will capture trends towards additivity and this strong non-additivity.

Here we build on a previous study of the relative involvement of three cortical regions in tactic selection and decision making in monkeys (Matsuzaka et al. 2012). That study focused solely on the post-stimulus activity to find differential correlates of action and tactic selection. The characteristics of the pmPFC activity stood out in terms of its involvement in tactic selection. It nevertheless behaved more similarly to the pre-SMA which is not surprising given the reciprocal connections between these areas. Both pmPFC and pre-SMA further differentiated themselves from the SMA, which has no direct connection to pmPFC. These areas do not specifically encode time-varying sensory stimuli, but do quickly respond to them within 200–$300~\text {ms}$.

Using both pre-and post-stimulus data from that same study, we demonstrate that a special type of strong non-additive interaction does occur at the single neuron level in primate cortex, especially in the pmPFC. This interaction is quantified by the difference in the time course of the firing rate as revealed by principal component analysis. It is also quantified via a comparison between two ratios computed for each neuron: (1) trial-to-trial changes in the ratio *Q* of pre- to post-stimulus spike counts, averaged individually across trials, and (2) the ratio *R* of the trial-averaged pre-stimulus spike count to the post-stimulus spike count. These two ratios differ in the order in which the averages are performed across trials. These statistics show that while many neurons appear to increase their trial averaged-rates upon stimulation, this is not the case at the single trial level in the area known to be most important (pmPFC) for the monkey’s choice of tactics and response during a decision making task. Thus we find that variability in the fine structure of single cell firing activity across the period of stimulus presentation differs between three areas involved in motor decisions. This represents a novel form of cellular non-additive interaction that provides an interesting counterpart to such interactions computed with macroscopic signals such as EEG or fMRI.

Our paper is organized as follows. The “Methods” section describes previously recorded data which we use in this paper, and the various analyses that we perform on them. The Results follow, paying attention first to the area-specific selection of responding neurons (heretofore referred to as “valid” neurons) via the Pearson correlation coefficient between spike counts pre- and post-stimulus. Then follows an analysis of the behavior of the aforementioned ratios *Q* and *R* in different areas, a principal component analysis to reveal the key differences in rate time courses across the three areas of interest and a surrogate data analysis to pinpoint the statistical nature of the firing process that exhibits non-additivity. The paper ends with a Discussion and Conclusion.

## Methods

### Stimulation protocol

The full experimental methods are described in Matsuzaka et al. (2012). Two monkeys (*Macaca fuscata*) were presented with red and green visual stimuli to perform a two-choice arm reaching task. A red stimulus signaled the monkey to reach for the right target, and a green stimulus to the left target. The task is made more challenging by the notion of *concordant* and *discordant* trials: in the former, the stimulus appears ipsilaterally to the target, whereas in the latter, it appears contralaterally to the target. In other words, the monkey has to reach towards the visual stimulus during concordant trials, and away from the visual stimulus during discordant trials. If the monkey is successful, it is rewarded with juice. For more details, we refer the reader to Matsuzaka et al. (2012).

**Fig. 1:**
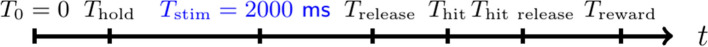
Timeline of events in the experiments from Matsuzaka et al. (2012). Recordings for each trial begin at $T_{0}$, the hold button is pressed at $T_{\mathrm {hold}}$, the cue stimulus is presented at $T_{\mathrm {stim}}$, the hold is released at $T_{\mathrm {release}}$, and the target button is hit at and released at $T_{\mathrm {hit}}$ and $T_{\mathrm {hit~release}}$, respectively. Finally, if the trial was successful, a reward is delivered at $T_{\mathrm {reward}}$. All times shown vary substantially from trial to trial with the exception of the stimulus onset time $T_{\mathrm {stim}}$. The stimulus onset time is in the middle of each recording, which is not depicted here according to scale due to space constraints

The data contain the timing of relevant behavioral events: hold onset, stimulus onset $T_{\mathrm {stim}}$, hold release $T_{\mathrm {release}}$, target hit, target release time and reward delivery. It further records for each trial the experimental layout and the outcome of the experiment, namely success or failure, depending on which button the monkey pressed. A timeline of these events is presented in Fig. 1.

Single cell firing activity was recorded in three areas of monkey cortex: the posterior dorsomedial prefrontal cortex (pmPFC), the supplementary motor area (SMA) and the presupplementary motor area (pre-SMA or pSMA). As was shown previously, the firing activity of neurons in the pmPFC is implicated in the choice of response tactics and in particular for the correct choice of response for the discordant trial type (Matsuzaka et al. 2012), in contrast to the two other motor-related regions. Moreover, we focus on the region pmPFC because neurons in this region were previously shown to be most active when choosing the response tactics for the dual-tactic task (both concordant and discordant trials are present) described above (Matsuzaka et al. 2012, 2016). This means that neurons in region pmPFC were more active when discordant and concordant trial types were presented in a random alternating fashion, i.e. when it was necessary for the monkey to select a response tactic. Their response time on concordant and discordant trials were statistically the same, suggesting that the animal developed tactics that compensated the cue-response conflict (Matsuzaka et al. 2012).

In summary, the work by Matsuzaka et al. (2012) suggests that the pmPFC neurons are less involved in action selection or monitoring response conflict. The distinction revolves around the fact that action selection is about deciding what to do, while tactic selection concerns how to go about deciding what to do. Tactic selection is linked to strategy and thus to supervisory control over motor behavior. In contrast, neurons in regions pre-SMA and SMA were equally active both under single-tactic and dual-tactic conditions. One of our goals is to see whether these three regions differ in the fine structure of single cell firing between the periods before and after the stimulus. This serves to complement the previous studies (Matsuzaka et al. 2012, 2016) which confined their focus only to the activity after the cue stimulus.

Given this finding of a graded response of different cortical regions in response to different behavioral challenges, and our expectation that the animal reaches a decision more easily in the concordant task, we split the ensemble of neural responses according to the cortical region and trial type (i.e. concordant or discordant). We will use the pre-SMA and SMA regions as a control for our findings in pmPFC. Unlike the original study (Matsuzaka et al. 2012) from which this data set was obtained, we will study the relationship between pre-stimulus and post-stimulus activity. In other words, we wish to understand how a given ensemble of neurons differentially reacts to a precisely timed stimulus, and whether certain regions can be distinguished based on features of these differential reactions.

### Selection of responding neurons

We mainly use the spike counts $N_{\text {pre}}$ and $N_{\text {post}}$ in suitably defined pre- and post-stimulus time intervals (see below) to quantify the neuronal activity pre- and post-stimulus. The principal component analyses (see below) were performed instead on the spike rates (spike count in a time window divided by the window length) which has the same information but in a temporally smoothed representation. The stimulus (go-cue) always occurs at $T_{\text {stim}} = 2000~\text {ms}$ (see Fig. 1). Only successful outcomes are considered below, as there weren’t many trials in which the monkeys pushed the wrong button. But our analysis accounts for the distinction between ‘Concordant’ and ‘Discordant’ trial types, so that we only consider data recorded under the mixed tactics condition, i.e. where both trial types were present.

The total numbers *n* of neurons (both ‘valid’ and ‘invalid’) in pmPFC, pre-SMA and SMA are 1268, 1266 and 1066, respectively. Data from two monkeys were pooled (one male and one female, for details see Matsuzaka et al. 2012). Each recording session corresponds to a number of trials on a given neuron; the number of such trials in each session is variable. We only consider trials with at least 6 spikes unless mentioned otherwise. There must be at least 3 spikes in both the pre- and post-stimulus periods. Our analysis does not differentiate between cue colors. Some neurons only have a low number of valid trials, which can result in nearly perfect correlation or anti-correlation of the spike counts in this trial. Therefore, we also excluded all neurons in which there were less than 4 trials for either the concordant or discordant trial type. In addition to requiring a minimal mean spiking activity of approximately $1.5~\text {Hz}$ (see above) in both pre- and post-stimulus intervals, only neurons for which both concordant and discordant trial types were recorded are considered here in order to facilitate comparison between trial types. This reduces the total number of neurons available.

Our study does not exclude time points, but rather considers the whole duration of pre- and post-stimulus time intervals unless mentioned otherwise. Specifically, the pre-stimulus time interval of duration $2000~\text {ms}$ is between $0~\text {ms} \le t < 2000~\text {ms}$, while the post-stimulus time interval of duration $2000~\text {ms}$ is between $2000~\text {ms} < t \le 4000~\text {ms}$. This choice is motivated by the fact that, since we only consider spike trains of a certain length, we wish to minimize the number of trials and neurons that are discarded from our analyses due to low spike counts. Another choice - not pursued here - would have been to look at post-stimulus intervals only up to the hold release time $T_{\text {release}}$, requiring in parallel an interval of length $T_{\text {release}}-2000~\text {ms}$ before the stimulus so that pre- and post-stimulus intervals have the same length. That choice is more reminiscent of a delay period in working memory tasks (Shafi et al. 2007). In either case, it is important that pre- and post-stimulus periods have comparable lengths, since otherwise the counting of spikes can be biased.

Responding or ‘valid’ neurons are chosen based on the Pearson correlation coefficient (PCC) $\rho$ computed on a plot between pre- and post-stimulus counts for each individual trial for each neuron. The significance level for our analyses was set at $\alpha = 0.01$. The PCC is positive (negative) when one variable shows a positively (negatively) sloped linear trend with respect to the other, and negative when the opposite is true. It is 0 when there is no linear trend. We also performed a two-sided t-test against $\rho = 0$ for the Pearson correlation coefficient. A significant deviation ($p<0.01$) of $\rho$ from zero means that the neuron is valid and thus kept for further analyses.

### Statistics of pre-post stimulus activity

The fine structure of the activity changes in neurons is assessed using two metrics. The first metric looks at the following average over all $N_{i}$ valid trials for a valid neuron *i*: $Q_{i} = \frac{1}{N_{i}}\sum _{i=1}^{N_{i}}\left( \frac{N_{\text {pre};i}}{N_{\text {post};i}} \right) = \langle \frac{N_{\text {pre}}}{N_{\text {post}}} \rangle _i.$ *Q* is larger than 1 if, on average, a neuron had a higher spike count $N_{\text {pre};i}$ before than after $(N_{\text {post};i})$ the stimulus onset, so that the neuron decreased its activity after the stimulus onset. In other words, *Q* reflects whether on average (across trials), a neuron increases its spike count ($Q < 1$) or decreases its spike count ($Q > 1$) in response to the stimulus.

The second metric looks at the ratio of the average pre-stimulus spike count to the post-stimulus spike count $R \equiv \frac{\langle N_{\text {pre}}\rangle _i}{\langle N_{\text {post}}\rangle _i}$, where the average is across all valid trials for a valid neuron. It differs from *Q* in the order of the averaging and ratio operations. For *R*, the relative size of pre-to-post-stimulus counts for one trial is not taken into account, but instead it focuses on the ratio of the separately computed and trial-averaged pre-and post-stimulus counts. *Q* is more informative to understand what the neuron typically did on a given trial. In the following, we omit the subscript trial index *i* in the averages for both *Q* and *R* for simplicity.

A large value for *Q* ($Q \ge 1$) together with a positive PCC $\rho >0$ indicates that the neuron decreased, on average across trials, its activity after the stimulus while still modulating its response as a function of pre-stimulus spike count. Because $\rho$ is positive, this means that increasing the pre-stimulus spike count for this neuron also increases the post-stimulus spike count on average.

There are a number of other analyses that we have performed but have not included because they did not reveal any significant differences between the areas. They include computing the pre-post change in the coefficient of variation of the interspike intervals, the pre-post change in the serial correlation coefficient of the interspike intervals and the Fano factor of the spike counts over different counting windows. We also investigated the slope of the pre-vs.-post spike count as it varies across successive short windows of the post-stimulus period, i.e. as it varies during the dynamic response to the cue, but did not find any significant inter-areal differences in this time evolution.

Concretely, for each trial, we computed the mean rate in the $500~\text {ms}$ preceding the stimulus and in 151 overlapping windows of width $\Delta =500~\text {ms}$ after the stimulus, moved in increments of $10~\text {ms}$. Thus, the first bin covers the interval $[2000~\text {ms}, 2500~\text {ms})$, the second bin the interval $[2010~\text {ms}, 2510~\text {ms})$, and so on. The last bin starts at $t = 3500~\text {ms}$. After obtaining these time-courses, regression slopes were computed across trials for each bin, resulting in a time-series of regression slopes of length 151. We repeated the same calculation for $\Delta = 1500, 300$ and $200~\text {ms}$. This did not yield any significant differences between the regions, but led only to a stereotypical monotonic decay as a function of time across all areas and for all trial types. Thus, whereas we will show that the three areas exhibit significant differences in the quantities that we have defined above, other statistical measures that we have not considered here may show significant differences between the three areas.

### Principal component analysis

This analysis was carried out to reduce the dimensionality of the responses across trials for all neurons, thereby exposing the main features of the rate variations caused by the stimulus. The analysis was done separately for each of the three cortical areas of interest. The dimensionality of the initial space was set to the number of valid neurons (for example, 235 neurons for pmPFC concordant in Fig. 3). Each dimension is the trial-averaged rate obtained from the spike trains using a Gaussian smoothing kernel with width $\sigma = 20~\text {ms}$ instead of $\sigma = 5~\text {ms}$ for the rates that were used for the surrogate analysis (see next section). Hence, the PCA analysis is performed on a matrix of trial-averaged rate time courses, one for each valid neuron for a given region (pmPFC, pre-SMA or SMA) and trial type (concordant or discordant). Each row of the matrix holds the trial-averaged rate time course for one neuron computed in $1~\text {ms}$ bins. There are $M = 4000$ bins. Each column holds the rate time course at one fixed time point for all *N* valid neurons (*N* differs between regions and trial types). Thus, the PCA data matrix has *M* columns and *N* rows. The dimensional reduction is now performed in the dimension of the rows to reduce the ensemble size, so-to-speak. This gives us a number of principal components, each of dimension *M*, together with the explained variances. PCA finds the directions (i.e. principal components or PCs) that successively comprise the most variance in the data. In Fig. 7, we plot one time course (PC1) or 2 time courses (PC1+PC2) separately for concordant and discordant trials and for each area, thus resulting in a huge reduction in the dimensionality of the firing activity across the neural ensembles.

### Surrogate point process model analysis

In order to understand the statistical nature of the spike count changes before and after the stimulus, we performed a surrogate analysis using a simple inhomogeneous Poisson process. The question we address with such a process is whether the changes observed in spike count statistics *R* and *Q* for each valid cortical neuron are expected when assuming that each neuron can be modeled as a Poisson point process with a time-dependent rate. We chose this rate neuron-wise as the trial-averaged rate. In each trial, the spike train is the sum of Dirac delta functions convolved with a smoothing kernel of standard deviation $\sigma = 5~\text {ms}$. We also tried $\sigma = 20~\text {ms}$ as for the PCA (see section above), but found that this choice is too coarse to generate any meaningful overlap between the experimental and the to-be generated surrogate data. Then, for each valid neuron, we use the surrogate model to generate 100 independent spike train realizations (which would correspond to individual trials per valid neuron) of the inhomogeneous Poisson process with the experimentally derived rate. This was achieved using a standard thinning algorithm (Lewis and Shedler 1979; Laub et al. 2015). We then computed $\langle N_{\text {pre}}\rangle$, $\langle N_{\text {post}}\rangle$, and the ratio *Q* and *R* of the trial-wise ratios as described above for the real data. We then compared the distribution of these quantities (one value for each quantity for a valid neuron) to the distribution of the corresponding quantity in the real data by means of a two-sided Kolmogorov–Smirnov (KS) test. We repeated this $M=100$ times to generate 100 surrogate distributions for *Q* and *R* and hence 100 *p*-values, whose distribution, along with their mean, we report below. Here, large *p*-values indicate that the distributions obtained from the surrogate analysis are similar to those found in the data. Smaller *p*-values indicate that the distributions are distinct and therefore, the surrogate analysis does not capture the data well.

## Results

### Quantifying pre-post spike count variations

Here we consider various spiking activity statistics (see “Methods”, “Statistics of pre-post stimulus activity” section). The single neuron quantities are $\rho$, *Q*, *R*, $\langle N_{\text {pre}}\rangle$ and $\langle N_{\text {post}}\rangle$. Each quantifies slightly different features of the spiking data. $\rho$ quantifies a trend in one neuron to either increase or decrease its post-stimulus activity as a function of the pre-stimulus activity.

On the one hand, *Q* directly quantifies whether the neuron, on average across trials, fired more before or after the stimulus on any given trial. Intuitively, for one neuron, *Q* in a scatterplot of post-stimulus spike count versus pre-stimulus spike count indicates whether points on average lie above ($Q < 1$) or below the identity line ($Q > 1$). Alternatively, for each neuron, *R* quantifies the ratio of its pre-stimulus to its post-stimulus counts, each averaged separately across all trials. This also tells us about whether the neuron increases or decreases its rate after the stimulus, but averages out the correlations between pre- and post-stimulus spike count fluctuations. Finally, $\rho$ tells us something about the shape of this cloud of points, namely how well it is fitted by a linear relationship, rather than its position in the scatterplot.

**Fig. 2:**
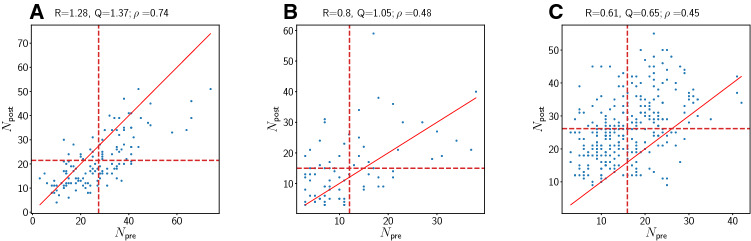
Examples for three possible scenarios for metrics *Q* and *R*. The data were taken from region pmPFC. Each panel shows ($N_{\text {pre}}$, $N_{\text {post}}$) pairs associated with a subset of trials for one valid neuron. The solid red line represents the identity line. The vertical and horizontal dashed red lines are the means of $N_{\text {pre}}$ and $N_{\text {post}}$, respectively. **a** $R>1$, $Q>1$. **b** $R<1$, $Q>1$. **c** $R<1$, $Q<1$. Case A corresponds to the case where the firing after stimulus onset decreases. Case B is representative of cases where *Q* and *R* convey different trends in the neuronal response to the stimulus. Thus, case B is a paradoxical case and of great interest. Case C corresponds to the common case where both *Q* and *R* indicate that the neuron has increased its firing after stimulus onset. Cases with $Q<1$, $R>1$ are not present in the data

We begin by considering the two main metrics *R* and *Q* whose typical behaviors are illustrated in Fig. 2. This figure illustrates three possible scenarios for combinations of *Q* and *R* that are present in the pmPFC data. Qualitatively similar figures are found for the other areas (not shown). This plot involves only neurons that are considered minimally responsive (which we call ’valid’), i.e. for which the value of $\rho$ determined from such a single-neuron plot is significantly different from zero (see below).

**Fig. 3:**
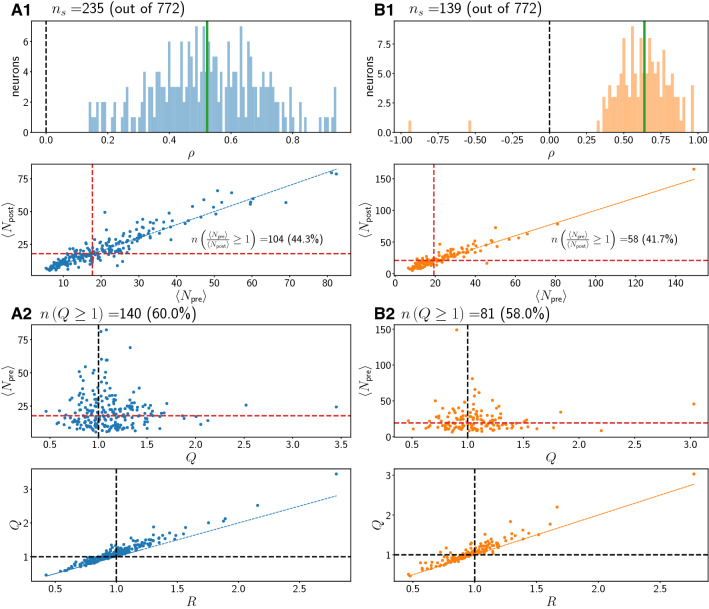
Statistics for region pmPFC. A1, A2: Concordant trials. B1, B2: Discordant trials. Red dashed lines denote medians of plotted quantities. Black dashed lines are at 1 or 0. Thin black dashed lines are regression fits. Solid blue (A1–A2) or solid orange (B1–B2) lines denote the identity line through the origin with slope 1 ($y=x$)

**Fig. 4:**
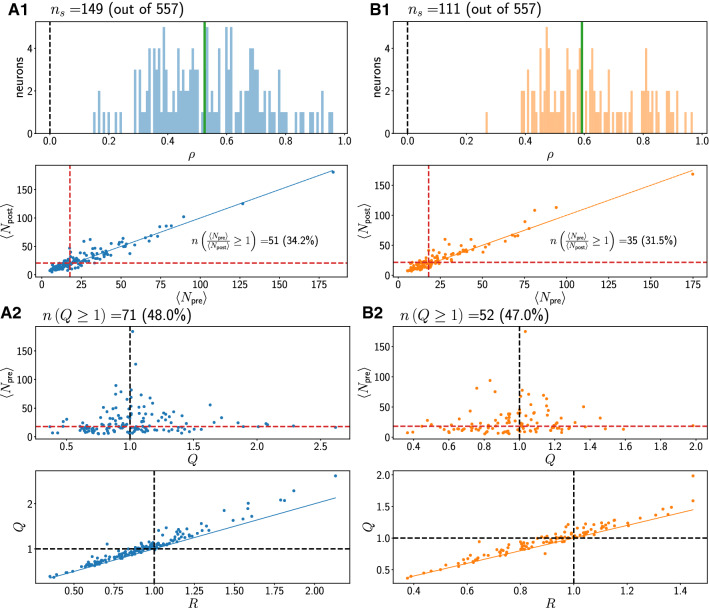
Statistics for region pre-SMA. A1, A2: Concordant trials. B1, B2: Discordant trials. Red dashed lines denote medians of plotted quantities. Black dashed lines are at 1 or 0. Thin black dashed lines are regression fits

**Fig. 5:**
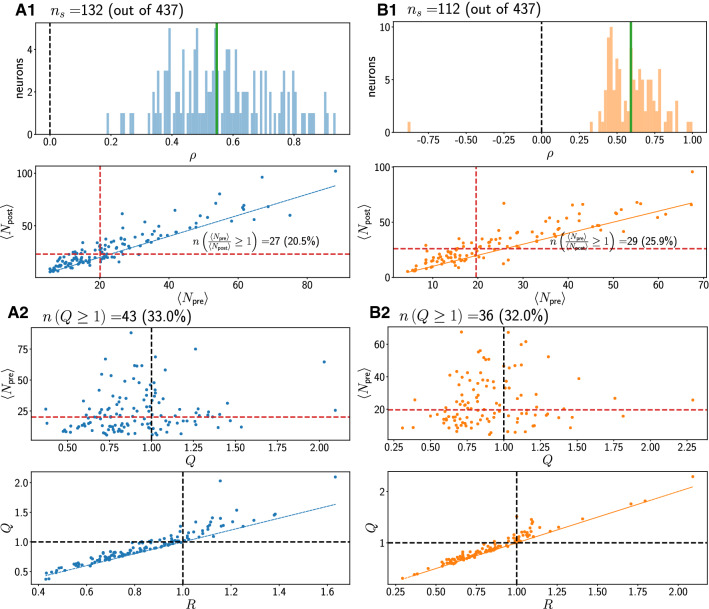
Statistics for region SMA. A1, A2: Concordant trials. B1, B2: Discordant trials. Red dashed lines denote medians of plotted quantities. Black dashed lines are at 1 or 0. Thin black dashed lines are regression fits

We first consider the variation of the spike count from the pre-stimulus to the post-stimulus period across trials, valid neurons and cortical areas. Our goal is to identify the fine structure of the trial-to-trial variability in the hope of extracting region-specific features. The minimal degree of response for a neuron to be labeled valid was determined using the Pearson correlation coefficient $\rho$ between pre- and post-stimulus spike counts, for every neuron separately across successful trials of one given trial type (concordant or discordant), for the three regions (pmPFC, pre-SMA and SMA) (see “Methods”, “Statistics of pre-post stimulus activity” section). In the following figures, we plot from top to bottom various statistics for concordant (Figs. 3, 4 and 5 A1, A2 in blue) and discordant (Figs. 3, 4 and 5 B1, B2 in orange) trial types separately for the three areas.

A1 panels show histograms (100 equally spaced bins) of the PCC $\rho$. This coefficient $\rho$ is calculated for a given neuron using all valid trials for that neuron. We show only the values of $\rho$ significantly different from 0, along with the total number of neurons that met this criterion. The majority of $\rho$ values are positive and scattered around the mean (vertical green line).

Also shown in A1 panels, below the histograms for $\rho$, is the mean (i.e. trial-averaged) post-stimulus spike count $\langle N_{\text {post}}\rangle$ as a function of mean pre-stimulus spike count $\langle N_{\text {pre}}\rangle$; each point in this plot corresponds to one neuron. We also write an inset with the number of neurons for which the pre-stimulus count was larger than or equal to the post-stimulus count as defined by the ratio *R* (see “Methods”, “Statistics of pre-post stimulus activity” section), i.e. which lie below the blue identity line. Most neurons show an increase in mean activity after the stimulus. There is thus a positive correlation between the mean spike counts before and after the stimulus when averages across trials are considered, i.e. when the detailed trial-to-trial variability is averaged out.

Moving down to panel A2 in Figs.  3, 4 and 5, we next show how the different single-neuron statistics correlate with one another. At the top of the panel, we indicate the number of neurons with $Q \ge 1$. The metric *Q* is larger than 1 for a neuron if, on average over its trials, there was a higher spike count before than after the stimulus onset, so that the neuron decreased its activity after the stimulus onset. *Q* is more informative to understand what the neuron typically did on a given trial. A large value for *Q* ($Q \ge 1$) together with $\rho >0$ indicates that the neuron decreased, on average across trials, its activity after the stimulus while still positively co-varying its response with pre-stimulus spike count.

We first show in panel A2 how $\langle N_{\text {pre}}\rangle$ correlates with *Q*. Little or no discernible correlation is visible. Next, in the second panel in A2, we show a scatterplot of the metrics *Q* and *R* for all valid neurons. *Q* and *R* are positively correlated. Most values for *Q* lie above the identity line, i.e. *Q* is larger than *R* for most neurons in all regions.

Comparing the top plots in A2 in Fig. 3 to those in Figs. 4 and 5, we see that in pmPFC, the majority of neurons have $Q \ge 1$ in both concordant and discordant trials. This is not the case in regions pre-SMA and SMA.

In B1 and B2, analogous plots for discordant trials are shown. The results for the statistics don’t differ markedly between concordant and discordant trial types in each region.

Summarizing the above we find that, on average and in all three regions, the post-stimulus count is higher if the pre-stimulus count is higher, i.e. a positive correlation $\rho$. Further, a majority of neurons have $R<1$, thus increasing their firing activity after stimulus onset. Concretely, the percentage of neurons that decrease their firing on average after the stimulus onset (as quantified by $R \ge 1$: see Figs. 3, 4 and 5 middle panels of A1 for concordant trials and B1 for discordant trials) is highest in pmPFC. For concordant (discordant) trials, these percentages are 44.3 % ($41.7 \%$), 34.2 % ($31.5 \%$) and 20.5 % ($25.9 \%$) in pmPFC, pre-SMA and SMA, respectively.

However, looking at the trial-resolved quantity *Q* reveals more information about the variability. Only in region pmPFC, most trials for the corresponding neurons show a decrease in firing after stimulus onset, i.e. the metric *Q* is equal to or exceeds 1 for most neurons. Specifically, the percentages of neurons for which $Q \ge 1$ are for concordant (discordant): 60 % ($58 \%$), 48 % ($47 \%$) and 33 % ($32 \%$) in pmPFC, pre-SMA and SMA, respectively, as shown in Figs. 3, 4 and 5 (see the top panels in A2 for concordant trials and B2 for discordant trials). The difference in *Q* between trial types in one region is not pronounced.

**Fig. 6:**
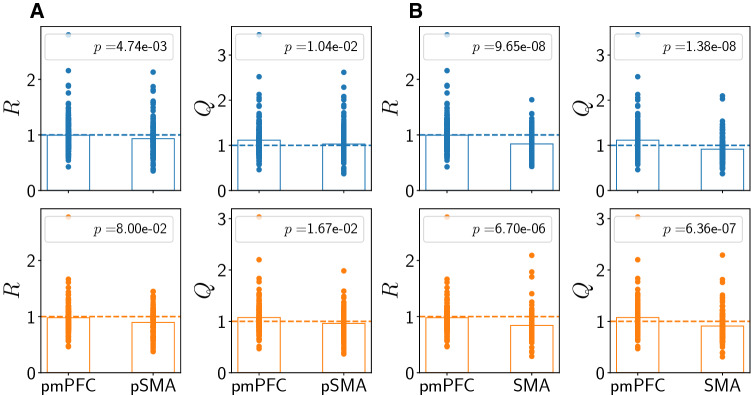
Comparison between regions. Top row: concordant trials, bottom row: discordant trials. **a**: Regions pmPFC and pre-SMA. **b**: Regions pmPFC and SMA. Vertical dashed lines are at 1. Bar heights denote means of the distributions, *p*-values are from two-sided two-sample KS tests. See also Supplementary Information, Figs. 1, 2, 3, 4 and 5

We show a summary for the regional differences of *Q* and *R* in Fig. 6. We quantify differences using two-sample two-sided Kolmogorov-Smirnov (KS) tests. The differences are clearest between the two regions pmPFC and SMA (Fig. 6b, where $p<10^{-5}$ for both *R* and *Q*). The differences between pmPFC and pre-SMA are not as pronounced and do not reach the $1 \%$ significance threshold (Fig. 6a). Given that we only analyzed trials from the mixed tactics condition, during which concordant and discordant trials are randomly presented, we expect from the results presented in Matsuzaka et al. (2012) that pmPFC and pre-SMA are more similar than pmPFC and SMA. Moreover, given that anatomically, the pmPFC and pre-SMA are reciprocally connected but the SMA lacks direct connection with the pmPFC, either afferent or efferent, we expect that the novel statistical measures that we exhibit are more similar between pmPFC and pre-SMA than between pmPFC and SMA. This is also confirmed in Fig. 6. The regional difference stems from the fact that more neurons have $Q>1$ (and to a lesser extent also $R>1$) in pmPFC than in SMA, but not in pre-SMA (cf. dashed horizontal lines in Fig. 6). These are neurons *decreasing* their spike count after cue onset. Thus, pmPFC differs from SMA and to a lesser extent also from pre-SMA because more neurons show stronger non-additive behavior on a single-trial basis, reflected in a larger proportion of neurons with $Q>1$.

We have added control analyses in the Supplementary Information (SI). In the SI, Fig. 1, we show results for the standard data set as in Fig. 6 for reference. In the SI, Fig. 2, we show results for shorter pre- and post-stimulus intervals. In the SI, Fig. 3, we show results when also spike trains with fewer spikes (1 instead of 3) in both pre- or post-stimulus interval are included. In the SI, Fig. 4, we show a combination: both fewer spikes per trial and shorter pre- and post-stimulus intervals are considered. The main finding is that the results shown in Fig. 6 remain valid except for short pre- and post-stimulus intervals when only neurons with at least 3 spikes in both pre- and post-stimulus intervals are included (SI, Fig. 2). In other words, shortening the pre- and post-stimulus intervals can be compensated for by allowing fewer spikes in each trial (SI, Fig. 4).

Next, we discuss the influence of the hold release time $T_{\text {release}}$ - a measure of behavioral latency - on the results in the SI, Fig. 5. To exclude that very large hold release times have an effect on our analysis, we show results where only trials with small hold release times ($T_{\text {release}}<2300~\text {ms}$) were considered (SI, Fig. 5). The results, although not as pronounced, are similar to the standard data set (Fig. 6); in particular, the differences between pmPFC and SMA remain significant at the $1\%$ level for all trials types and both *Q* and *R*. Even when only keeping trials with hold release times smaller than or equal to $2250~\text {ms}$, the structure of the results still holds (not shown): differences between pmPFC and SMA for *Q* are significant ($p<10^{-3}$), whereas differences between pmPFC and pre-SMA are not significantly different ($p>10^{-2}$).

Therefore, we conclude that the behavioral latency results, as quantified by the hold release time statistics, do not invalidate, and in fact lend support to our main conclusions presented in Fig. 6. We further conclude that our findings of regional differences for *Q* and *R* remain valid across a broad range of data inclusion choices.

### Principal components of firing rate time courses

We next asked whether pre- and post-stimulus activity features can be qualitatively distinguished region-wise and condition-wise by the trial-averaged time courses of the firing rates. This would provide a finer-grained representation of the neural activity, in contrast to the coarser-grained spike counts over longer windows, i.e. to the very coarse-grained firing rate picture considered up to now. To address this question, we computed a low-dimensional representation of the firing rate time course from all valid neurons for a region and condition, namely, the principal components of the rate time courses (see “Methods”, “Principal component analysis” section).

**Fig. 7:**
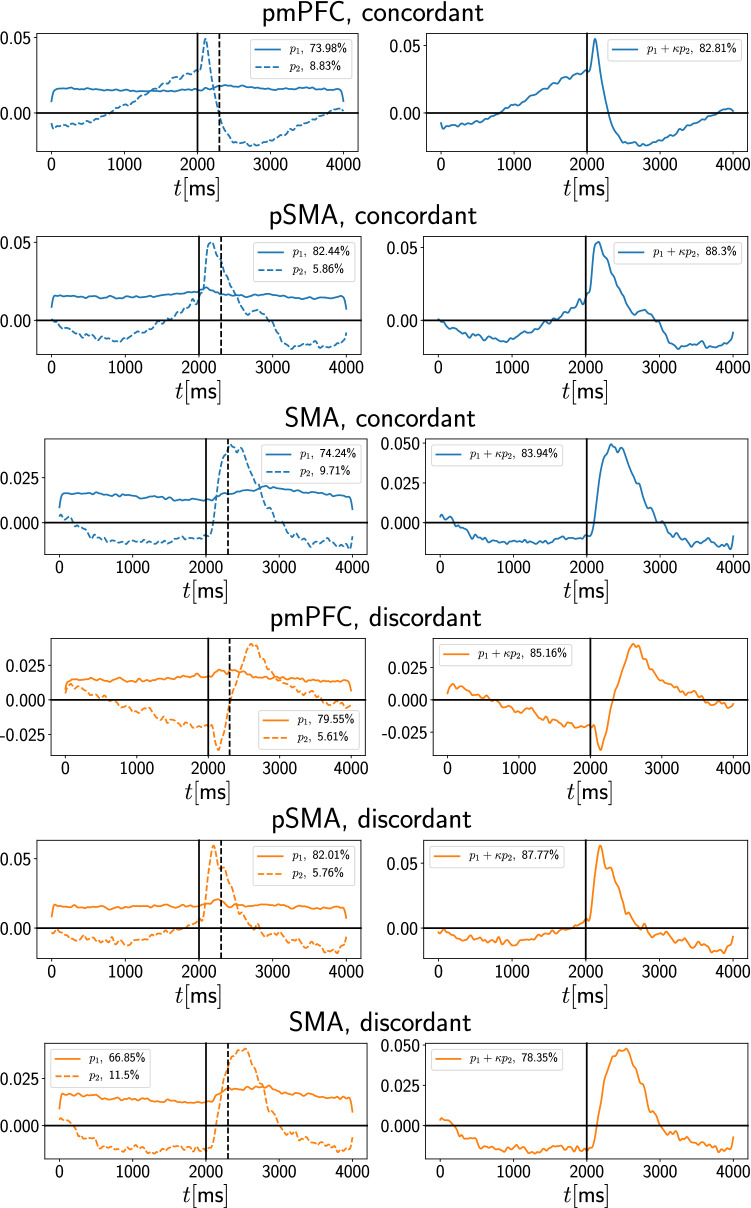
PCA analysis. For each response type and region of interest, the firing rate time course to which the PCA analysis is applied is obtained by averaging across all relevant trials for one valid neuron. Top three panels (blue lines) are for the concordant trial type, while the bottom three panels (orange lines) are for the discordant trial type. Left: First and second PCs together with their explained variance. Right: Weighted sum of first and second PC. The weighting factor $\kappa$ is given by ratio of the explained variance of the second and first principal component, whose values are indicated in the legend of the left subplot

The rate time course for each trial was computed using a Gaussian window of width $\sigma = 20~\text {ms}$. The first two principal components together explain more than $80 \%$ of the variance for concordant trials, and around $80 \%$ for discordant trials (Fig. 7). The third and higher PCs each carry less than $10 \%$ of the total variance. For concordant trials in pmPFC, the second PC ramps up before the onset of the stimulus. In pre-SMA for concordant trials, the second PC shows less ramping-up behavior, which is reflected in the weighted sum of PC1 and PC2.

Importantly however, the difference between concordant and discordant PCs is most pronounced in region pmPFC. The ramping behaviour present during concordant trials is not displayed during discordant trials. Further, region pmPFC shows markedly different trial-averaged rate time-courses compared to the other two regions. The pre-stimulus activity is monotonic for pmPFC concordant, which contrasts with a non-monotonic behavior in all other areas and conditions where the baseline activity first dips and then ramps up again and beyond the cue.

Finally, we now explore whether the trial-averaged data whose dynamics we described above can be used in a modeling context to reproduce the statistics of non-additivity described in the two previous sections.

### Surrogate spike trains

**Fig. 8:**
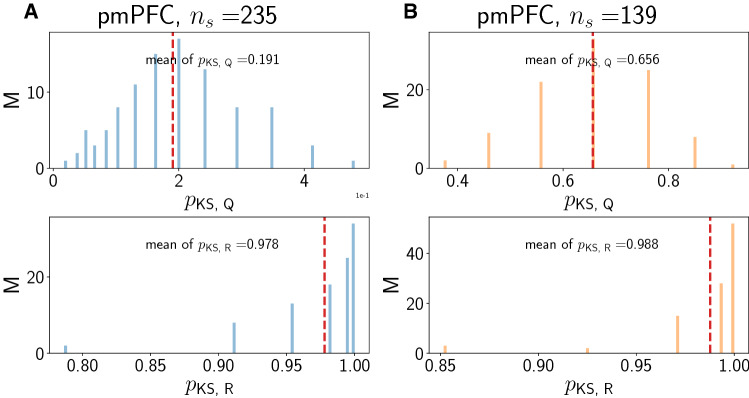
Surrogate data for region pmPFC. A/B: Concordant/discordant trials. Distribution of *p*-values from two-sided KS test for *Q* (top) and *R* (bottom). In each panel, the red dashed vertical line denotes the mean of the distribution

**Fig. 9:**
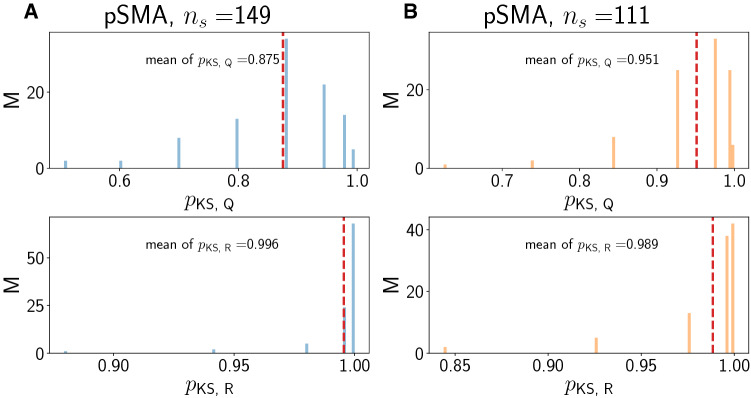
Surrogate data for region pre-SMA. A/B Concordant/discordant trials. Distribution of *p*-values from two-sided KS test for *Q* (top) and *R* (bottom). In each panel, the red dashed vertical line denotes the mean of the distribution

**Fig. 10:**
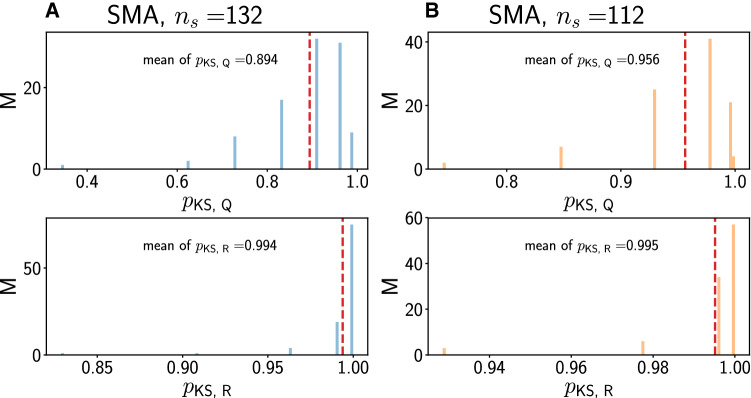
Surrogate data for region SMA. A/B: Concordant/discordant trials. Distribution of *p*-values from two-sided KS test for *Q* (top) and *R* (bottom). In each panel, the red dashed vertical line denotes the mean of the distribution

Results with the surrogate point process model (see “Methods”, “Surrogate point process model analysis” section) are presented in Figs. 8, 9 and 10. An inhomogeneous Poisson process rate is fitted to the trial-averaged response for each neuron based on all spikes, rather than on the principal components discussed in the preceding section. In spite of different amounts of smoothing, namely for kernel widths $\sigma = 5~\text {ms}$ for the surrogate analysis instead of $\sigma = 20~\text {ms}$ for the PCA, the rate time courses are the same for the surrogate analysis and the PCA.

We compute *Q* and *R* from 100 trials (independent samples from the trial-averaged rate) for each valid neuron as described in “Methods”, “Surrogate point process model analysis” section. Given that *R* is a trial-averaged quantity, we expect that it is always reproduced well by the surrogate analysis. This is confirmed by the large *p*-values close to 1 in the bottom panels of Figs. 8, 9 and 10. For *Q* however (top panels in Figs. 8, 9 and 10), the results are markedly different. The *p*-values are smaller than for *R* across regions, indicating that the surrogate analysis does not capture the distribution for *Q* as well as for *R*. For pmPFC concordant, the smallest mean *p*-value is obtained, followed by pmPFC discordant. Mean *p*-values for *Q* in pre-SMA and SMA are larger than in pmPFC, but smaller than for *R*. This indicates that the surrogate analysis for *Q* works slightly better in pre-SMA and SMA than in pmPFC, but worse than for *R*. Thus, non-additivity as we have defined it is also reflected by how well trial-averaged rates approximate single-trial quantities.

We note that the fraction of neurons that have $R \ge 1$ and $Q \ge 1$ is approximately reproduced by the surrogate analysis. For pmPFC, neuron numbers with $Q \ge 1$ for concordant/ discordant are 133.34/73.11 versus 140/81 in the data. For $R\ge 1$, the corresponding numbers are 101.34/60.46 versus 104/58. Similar observations hold for the regions pSMA and SMA (pSMA: $n(Q \ge 1)$: 65.92/48.08 versus 71/52, $n(R\ge 1)$: 51.7/36.15 versus 51/35; SMA: $n(Q \ge 1$): 39.31/36.17 versus 43/36, $n(R\ge 1)$: 27.89/27.52 versus 27/29).

We conclude that *Q* and *R* are related (see also the *Q*-*R* scatterplots in the lower parts of panel A2 and B2 in Figs. 3, 4 and 5), but the surrogate analysis clearly only captures the behavior of *R* and not of *Q* across all regions and trial types. The agreement for both *Q* and *R* is better in SMA than in pre-SMA and pmPFC. The agreement for *R* is only slightly better in pre-SMA than in pmPFC.

## Discussion

### Summary and implications

We have presented a study of the single cell firing statistics and a model for such statistics in different cortical areas related to motor tactics and decision making. Our goal was first to see whether non-additivity effects are at work in these areas in shaping the pre- to post-stimulus transition in cortical firing activity. We have uncovered a strong form of non-additivity that manifests itself at the single trial level. Only in region pmPFC, it involves a slight majority of neurons decreasing their spike count in a post-stimulus window in comparison to a window of same duration immediately before the stimulus. The associated statistic *Q* takes into account the trial-to-trial variability of pre- and post-stimulus spike counts, as well as the trial-wise covariations of these counts. This slight majority turns into a slight minority when the spike counts forming the pre-to-post count ratio are first averaged across trials, which removes the correlated variability seen trial-to-trial. This is the case in pmPFC for both concordant and discordant trial type, and supports previously reported studies that this area is the main player in the tactic selection and decision process (Matsuzaka et al. 2012).

Hence, in pmPFC, the time courses of neural activity before and after the stimulus show marked variability and both increases and decreases in mean firing rate. In the two remaining motor-related regions pSMA and SMA, this is not as strongly the case: neurons more generically just increase their firing rates after stimulus onset. This complex modulation of neuronal activity in pmPFC might serve as a neural correlate for successful tactic selection for decision making during a two-choice forearm reaching task. These results speak to the importance of paying attention to the trial-to-trial statistics as well as to the heterogeneity of responses across cell types in regions underlying cognitive behaviour. Thus, they support the view put forward by Shafi et al. (2007).

We have presented evidence for non-additive interactions between spontaneous and evoked activity on the cellular level of the single neuron including its association with behavioral performance. Together with recent data on the regional level of neural activity (He 2013; Huang et al. 2017; Wainio-Theberge et al. 2021), our results put the generality of the commonly assumed additive model for the relation between spontaneous and evoked activity into doubt. This, as also demonstrated in our data, carries major implications for assessing the relationship between stimulus-induced activity and behavior.

The analysis of the experimental data point to the fact that the statistic *Q*, in which the numerator and denominator are evaluated on a trial-to-trial basis, differ from those using an average of $N_{\text {pre}}$ and $N_{\text {post}}$ across trials, which forms the basis of the statistic *R*. In parallel, the CV of spike counts systematically increases after the cue (not shown). The correlated fluctuations in the relative size of pre-and post-stimulus counts for individual trials are washed away in the averaging operation. Trials that see a large increase in spike count after stimulus onset can therefore dominate and tilt the results to a ratio $R=\frac{\langle N_{\text {pre}}\rangle }{\langle N_{\text {post}}\rangle }$ that can still be less than one for a given neuron. While this is not a strong effect, it is significant (Fig. 6). It may be related to the manner in which we have estimated the rate for each neuron, but it is nevertheless a property of an inhomogeneous process with the rate as we computed it with a standard smoothing kernel (“Methods”, “Surrogate point process model analysis” section and Figs. 8, 9 and 10).

The neural ensemble where most neurons have $Q \ge 1$ is thus only present in region pmPFC. In fact, the size of the neural ensemble with $Q \ge 1$ monotonically decreases from pmPFC to pre-SMA to SMA (Figs. 3, 4 and 5). Why might such an ensemble endow the monkey with a neural basis for cognitive processes that leads to fast and successful decision making in the presence of a cue-response conflict? Whereas the answer of this question is well beyond the scope of the present study, *we speculate that such non-additive subensembles function as a neural correlate for mental preparation in anticipation of a stimulus*, that in the experiments analyzed here takes the form of a go-cue, prompting the well-trained monkey to press either a left or right button. Differently put, we hypothesize that the non-additive sub-ensemble sets the state for mental processes that then lead to the tactic selection and ultimately successful behavior in the form of correct button presses. Answering this question would require a selective silencing of the non-additive subensemble.

Different mechanisms may underlie the observations of strong non-additivity that our analyses reveal. They can be supported by special single cell input-output functions and/or by circuitry effects involving differential responses of E and I neurons, but pinpointing this mechanism is not possible given the available experimental data. The cue likely changes the E-I balance of inputs to the neuron under consideration. But not knowing whether a given neuron is of E or I type, along with the notorious complexity of cortical circuitry with E cells and different types of I cells (some of which inhibit each other), impedes the narrowing down of possibilities. One can speculate, e.g. by assuming that a strongly non-additive cell with both $R>1$ and $Q>1$ is an E cell. Its activity will decrease if it receives more inhibition and/or less excitation, i.e. its net input decreases following the cue. This suggests that neurons whose activities go down are E cells; it would be less likely for the activity of pre-synaptic I cells to go down in this scenario, unless this decreased inhibition were counteracted by an even larger decrease in excitation from the network following the cue. A similar story can be elaborated in the case where the recorded cell is of I-type. A more subtle possibility could implicate one inhibitory population I1 inhibiting another inhibitory population I2. Then, if the cue increases the activity of I1, the activity of I2 would drop, and a recording of I2 would be interpreted as strong non-additivity due to increased inhibition. An alternate scenario would see the cue somehow decrease the activity of I1, thus raising that of I2, thus decreasing that of the E cells. In this scenario, the strong non-additivity would be seen directly in I1, as well as in the E cell as a consequence of disinhibition of I2.

Only more detailed modeling of an EI network with different hypotheses for the effect of a “cue” input could make predictions about the outcomes, and this would entail many control simulations. This is also the case for the more subtle situations where $Q>1$: the Poisson neurons reproduce some of the statistics well, except for pmPFC concordant. Then, all that we can say is that these pmPFC neurons, whatever their internal firing dynamics and inputs are, behave the least like inhomogeneous Poisson neurons. This may be due to network effects, i.e. the changes in the net input to the cell. But this may also arise in part from distinct deviations of pmPFC single neuron firing properties from generic Poisson spiking, e.g. in the form of nonlinearities and adaptation which will add correlations between firing events with, and even without a stimulus (Braun et al. 2017).

The surrogate analysis (Figs. 8, 9 and 10) further enabled us to show that only the trial-averaged quantity *R* can be convincingly modeled by an inhomogeneous Poisson process whose time-dependent rate has been fitted from the individual trials for that neuron. For *Q*, which reflects more reliably what the neuron actually did on a given single trial, the surrogate analysis breaks down in region pmPFC, especially for concordant trials. It is more reliable in pre-SMA and further still in SMA. Thus, this surrogate analysis enables the distinction between the three areas. One can argue that the surrogate-based distinction between the concordant and discordant cases for pmPFC shows that the statistics employed by these neurons are most altered under the two experimental conditions, and that tactic selection may somehow utilize - or impose - these different firing patterns. This also speaks to the previously reported distinction between pmPFC and the pre-SMA and SMA with respect to decision making.

More detailed modeling is required to better reproduce, and thus understand, the response variability seen for concordant pmPFC and the other areas and trial types. For now we can also add that the surrogate model can reproduce similar pre- and post- stimulus spike counts, but not their ratio on a single-trial basis. Since the simple inhomogeneous Poisson model can’t account for the behavior of *Q*, it is likely that more assumptions, i.e. mathematical features, need to be incorporated in the model. These features could offer more insight into the origin of the finer statistical structure of the pre-to-post activity changes (see discussion on “Methodological limitations” section below).

A further possibility that we considered in devising a surrogate analysis would be to perform a rate estimate for each trial for each neuron. Then we could compute the statistics *Q* and *R* anew for each trial from multiple realizations of the resulting inhomogeneous Poisson process. We felt however that, although this approach would likely lead to better fits to the observed statistics, it would amount to an over-fitting of the data from which little could be learned. Future work could focus on unraveling the characteristics of the trial-averaged rate time course that lead to the observed statistics.

There is much interest in the origins of the variability in evoked cortical responses at the cellular level, dating back at least to the seminal work of Arieli et al. (1996) and Azouz and Gray (1999). Later studies have quantified the various types of interactions between spontaneous and evoked activity including non-additive interactions ((He 2013; Northoff et al. 2010; Huang et al. 2017). The last decade has seen efforts to pin down contributions from various mechanisms that prepare the state of the system such as oscillatory tendencies (Haslinger et al. 2006; Curto et al. 2009), or memory of past stimuli (Marcos et al. 2013). It continues to this day with dynamical modeling studies of neural activity that consider the impact of the stimulus on the phase space trajectories available to the system (Ponce-Alvarez et al. 2015). A recent study has even predicted how the variability of cortical activity due to the chaotic motion combined with stochastic synaptic transmission is compatible with a temporal code for spikes in response to a deterministic input (Nolte et al. 2013). It remains to be seen whether there are further distinctions between areas and conditions that could be made in our data on the basis of temporal coding rather than spike counts. Headway into this question could build on such modeling efforts involving more biophysically realistic cellular and synaptic dynamics.

Our PCA analysis in Fig. 7 reveals that the time course of the two main components capture around 80% of the variance of the data in all regions and for both trial types. Interestingly, the PC1+PC2 activity exhibits little qualitative difference between the concordant and discordant conditions for both the pSMA and SMA regions, but this is not the case for the pmPFC region. In contrast, two features stand out from the pmPFC results.

Firstly, the concordant and discordant conditions differ both before and after the stimulus. Prior to the stimulus, the concordant stimulation sees a monotonic increase in PC activity, while the discordant condition displays a non-monotonic activity. Post-stimulus, the concordant condition has a bi-phasic character, exhibiting a narrow peak followed by a trough. This differs from the discordant condition which first dips and then has a broad peak followed by a monotonic decrease. Secondly, the behavior of the pre-stimulus period is monotonic for pmPFC concordant, while for all other areas and conditions, a non-monotonic behavior appears.

The obvious difference in the shape of the PC responses between pmPFC and the other regions supports the distinctive character of this region in terms of its implication in tactic selection and decision making. The same can be said regarding the salient difference between PC responses in the pmPFC concordant and discordant cases. This cortical area is clearly at work producing separate response features for separate trial types.

Overall, pmPFC behaves differently than the other two areas with respect to the statistics of *Q*, *R* and the PCA. Furthermore, in the surrogate analysis, pre-SMA and SMA show more similar results than pmPFC for *Q*. Thus, our analysis using pre-post stimulus statistics further supports the special role pmPFC plays in terms of tactic selection.

### Methodological limitations

The fact that the simplest inhomogeneous Poisson process model does not account for the pre-post changes in firing statistics for *Q* in all areas (although the agreement for SMA and pre-SMA is clearly better than for pmPFC, Figs. 8, 9 and 10) raises interesting questions about the cellular and network dynamics that underlie firing in those cases. We have considered only inhomogeneous Poisson processes with zero deadtime, i.e. with no absolute refractory period following a spike. While refractory effects may not be expected at low firing rates, certain cortical neurons nevertheless can exhibit threshold increases or other adaptive properties following spiking, making the Poisson spiking with time-dependent rate picture potentially too simplistic. Pushing the analysis in this direction could enhance the agreement between the firing behaviors of the surrogate model and those observed in the data, leading perhaps to more distinctions between the areas and conditions, and providing more precise targets with which to eventually validate network models of those areas.

Ultimately, repeating the experiments over the same areas but with optogenetically tagged neurons would enable a parsing out of the effects related to neural subtypes, e.g. to principal cells versus interneurons and associated circuitry. Finally it may be that there is a story behind the neurons that do not significantly change their firing rate based on our Pearson correlation coefficient criterion and which we therefore excluded from our analysis. Other statistics may reveal responsiveness and in turn this may alter the conclusions of our study.

## Conclusion

In conclusion, we here demonstrate a novel form of strong non-additivity in the interaction between pre-stimulus and post-stimulus activity on the cellular level of neuron ensembles in the medial prefrontal cortex of macaque. This non-additivity highlights that a majority of trials see spike counts decrease following a cue stimulus, in contrast to the ratio of pre-to-post trial-averaged counts. Our data show additive and non-additive sub-ensembles, the proportions of which vary between the regions and the concordant versus discordant trial type. The temporal dynamics of firing of the non-additive sub-ensemble in pmPFC, and associated fine structure of the firing statistics, stands apart from that in the other regions. This further supports the distinctive nature of pmPFC activity and its closer association with decision making in motor tasks involving multiple tactics as reported in previous studies based solely on post-stimulus statistics (Matsuzaka et al. 2012, 2016). A surrogate data analysis reveals that the behavior of the trial-averaged quantity *R* is well modeled by an inhomogeneous Poisson process in which the time-dependent firing rate is the neuron-specific trial-averaged rate. Our data at the cellular level complement recent results on the regional level with fMRI and extend the relevance of non-additivity of rest-stimulus interactions to complex behaviors relating to decision making.

## Supplementary Information

Below is the link to the electronic supplementary material.

Supplementary file1 (PDF 304 kb)

## Data Availability

The data is available from Y.M. upon request.

## Code availability

The custom analysis code is available from the corresponding author upon request.
