# Supplementary material for: Non-additive activity modulation during a decision making task involving tactic selection
Source: Cogn Neurodyn. 2021 Jul 30;16(1):117–33. doi: 10.1007/s11571-021-09702-0 (PMC8807796; doi:10.1007/s11571-021-09702-0)
Supplement: Supplementary file 1 — Supplementary file1 (PDF 304 kb) [file 11571_2021_9702_MOESM1_ESM.pdf]

Supplementary information for submission CODY-D-20-00182

W. Braun, Y. Matsuzaka, H. Mushiake, G. Northoff and A. Longtin

April 6, 2021

Figs. 1 - 5 below refer to Fig. 6 in the main manuscript.

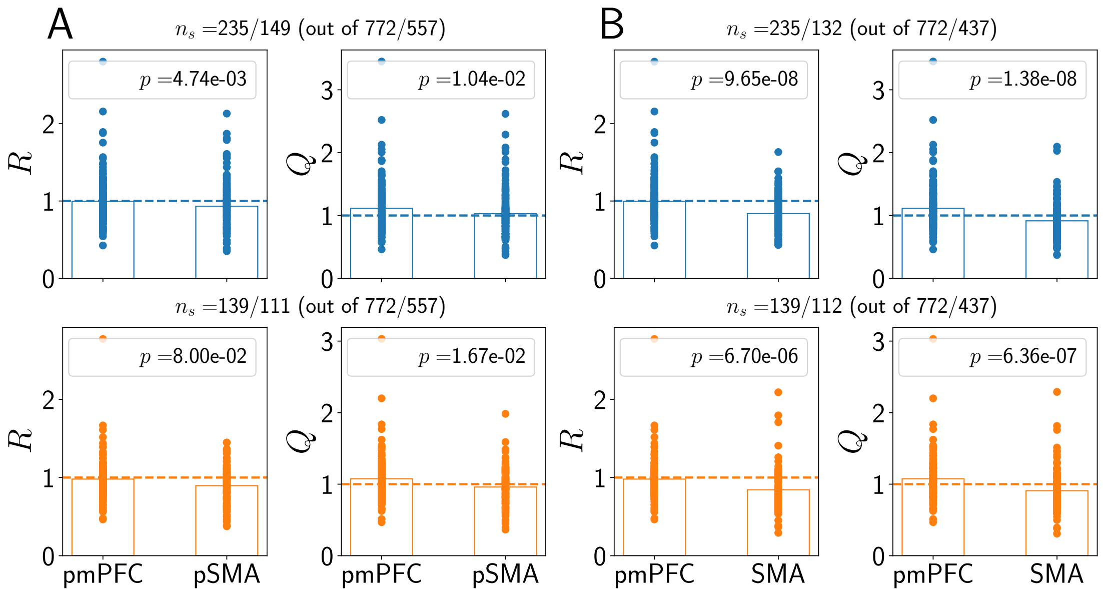

Figure 1: **Comparison between regions for standard data set** (equivalent to Fig. 6 in the main manuscript, but reproduced here for ease of comparison). Top row: concordant trials, bottom row: discordant trials. A: Regions pmPFC and pre-SMA. B: Regions pmPFC and SMA. Vertical dashed lines are at 1. Bar heights denote means of the distributions, p-values are from two-sided Kolmogorov-Smirnov (KS) tests.

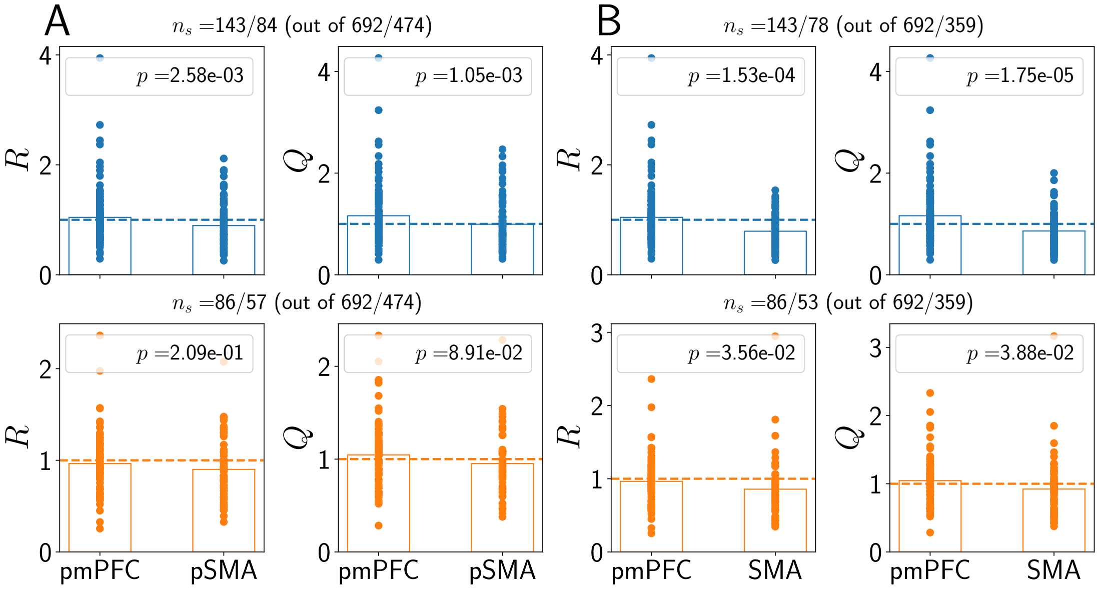

Figure 2: **Comparison between regions using shorter pre- and post-stimulus intervals.** Top row: concordant trials, bottom row: discordant trials. A: Regions pmPFC and pre-SMA B: Regions pmPFC and SMA. Vertical dashed lines are at 1. Bar heights denote means of the distributions, p-values are from two-sided KS tests. In comparison to Fig. 1, the length of pre- and post-stimulus intervals have decreased, such that the pre-stimulus interval extends from 1000 to 2000 ms (instead of from 0 to 2000 ms) and the post-stimulus interval from 2000 to 3000 ms (instead of from 2000 to 4000 ms). Because we still enforce the minimum of 3 spikes in both the pre- and post-stimulus intervals, there are less valid neurons than in the standard set. The shortening of the intervals results in less pronounced differences between the regions; only concordant trials (top panels) significantly differ at the 1% level. Our original analysis involving longer pre- and post-stimulus periods thus better revealed differences.

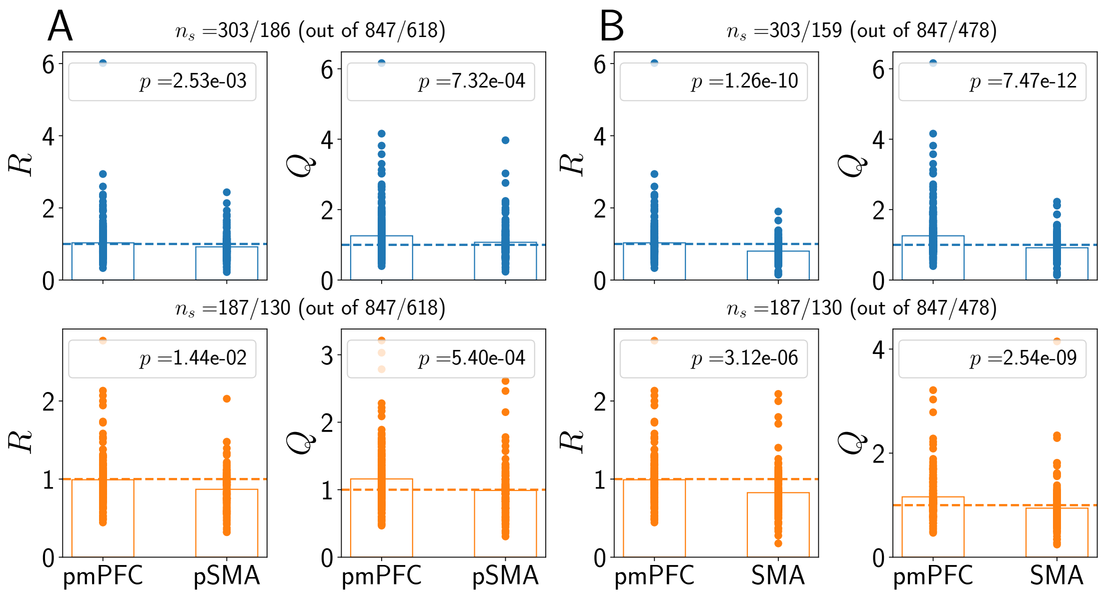

Figure 3: **Comparison between regions using fewer spikes to select valid neurons** (1 instead of 3 in each pre- and post-stimulus interval). Top row: concordant trials, bottom row: discordant trials. A: Regions pmPFC and pre-SMA. B: Regions pmPFC and SMA. Vertical dashed lines are at 1. Bar heights denote means of the distributions, p-values are from two-sided KS tests. Spike trains have at least one spike (in contrast to three spikes for the standard data set, Fig. 6 in the main manuscript) in both the pre- and post-cue interval. This increased the number of neurons that our analyses were applied to (see subfigure headings). This looser selection criterion, compared to our minimum of 3 spikes pre- and post-cue, results in more significant differences between pmPFC and SMA for both concordant and discordant trials (panel B).

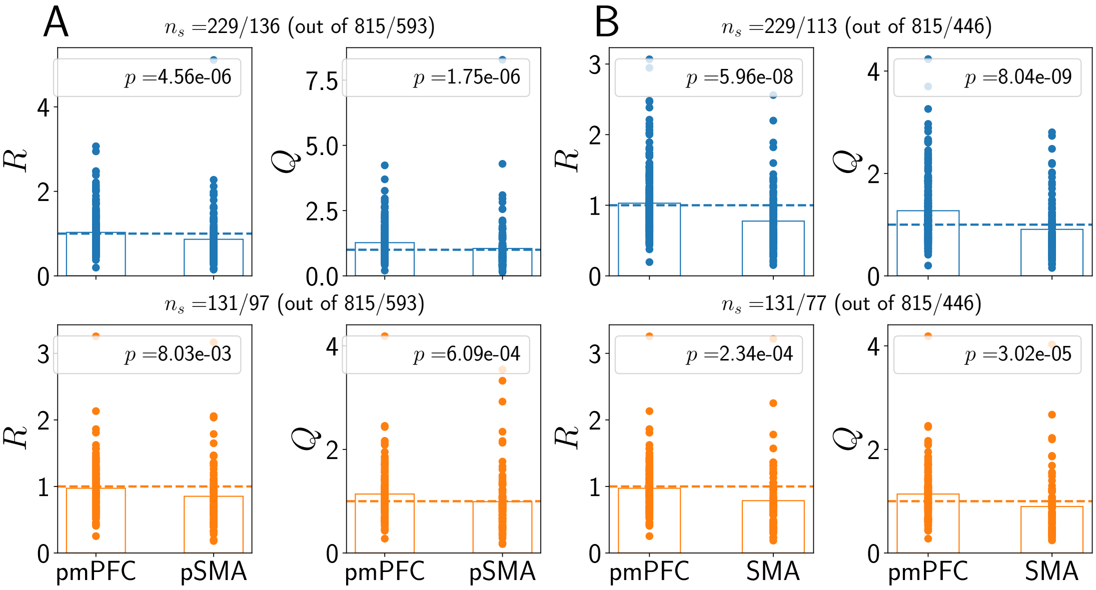

Figure 4: **Comparison between regions using shorter pre- and post-stimulus intervals** (as in Fig. 2) as well as fewer spikes (1 instead of 3 in each pre- and post-stimulus interval as in Fig.3) to select valid neurons. Top row: concordant trials, bottom row: discordant trials. A: Regions pmPFC and pre-SMA. B: Regions pmPFC and SMA. Vertical dashed lines are at 1. Bar heights denote means of the distributions, p-values are from two-sided KS tests. Both the durations of pre- and post-stimulus intervals are decreased and the minimal number of spikes per interval is decreased. The differences between pmPFC and SMA, but also those between pmPFC and pre-SMA, become significant at the 1% level. Importantly, the p-value for Q is always slightly smaller than the p-value for R when differences are significant, such that Q yields a more informative measure than R to distinguish between regions and trial types.

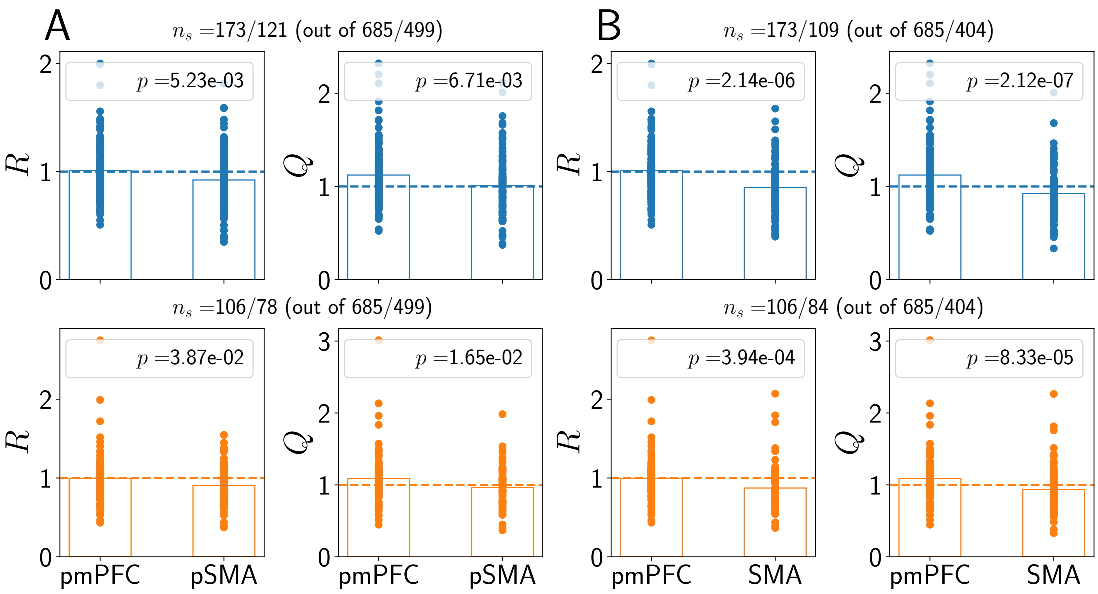

Figure 5: **Comparison between regions when only trials with hold release times smaller than or equal to** 2300 **ms are considered.** Top row: concordant trials, bottom row: discordant trials. A: Regions pmPFC and pre-SMA. B: Regions pmPFC and SMA. Vertical dashed lines are at 1. Bar heights denote means of the distributions, p-values are from two-sided KS tests. To exclude that very large hold release times have an effect on our analysis, we show results where only trials with small hold release times ($T_{\text{release}} < 2300$ ms) were considered. The results, although not as pronounced, are similar to the standard data set (Fig. 1 above); in particular, the differences between pmPFC and SMA remain significant at the 1% level for all trial types and both Q and R.
